# An Intracellular Transcriptomic Atlas of the Giant Coenocyte *Caulerpa taxifolia*

**DOI:** 10.1371/journal.pgen.1004900

**Published:** 2015-01-08

**Authors:** Aashish Ranjan, Brad T. Townsley, Yasunori Ichihashi, Neelima R. Sinha, Daniel H. Chitwood

**Affiliations:** 1 Department of Plant Biology, University of California at Davis, Davis, California, United States of America; 2 Donald Danforth Plant Science Center, St. Louis, Missouri, United States of America; The University of North Carolina at Chapel Hill, United States of America

## Abstract

Convergent morphologies have arisen in plants multiple times. In non-vascular and vascular land plants, convergent morphology in the form of roots, stems, and leaves arose. The morphology of some green algae includes an anchoring holdfast, stipe, and leaf-like fronds. Such morphology occurs in the absence of multicellularity in the siphonous algae, which are single cells. Morphogenesis is separate from cellular division in the land plants, which although are multicellular, have been argued to exhibit properties similar to single celled organisms. Within the single, macroscopic cell of a siphonous alga, how are transcripts partitioned, and what can this tell us about the development of similar convergent structures in land plants? Here, we present a *de novo* assembled, intracellular transcriptomic atlas for the giant coenocyte *Caulerpa taxifolia*. Transcripts show a global, basal-apical pattern of distribution from the holdfast to the frond apex in which transcript identities roughly follow the flow of genetic information in the cell, transcription-to-translation. The analysis of the intersection of transcriptomic atlases of a land plant and *Caulerpa* suggests the recurrent recruitment of transcript accumulation patterns to organs over large evolutionary distances. Our results not only provide an intracellular atlas of transcript localization, but also demonstrate the contribution of transcript partitioning to morphology, independent from multicellularity, in plants.

## Author Summary

Plants include both the green algae and land plants. Multiple times, root, stem, and leaf-like structures arose independently in plant lineages. In some instances, such as the siphonous algae, these structures arose in the absence of multicellularity. It has been argued by some that the morphology of multicellular land plant organs similarly arises independently of cell division patterns. Here, we explore the partitioning of gene transcripts within what is debatably the largest single-celled organism in the world, the siphonous alga *Caulerpa taxifolia*. We find that within this giant cell specific transcripts localize within pseudo-organs (morphological structures that are not comprised of cells or tissue). The overall pattern of transcript accumulation follows an apical-basal pattern within the cell. Moreover, transcripts related to different cellular processes, such as transcription and translation, localize to specific regions. Analyzing the signatures of transcript accumulation in land plant organs and the pseudo-organs of *Caulerpa*, we find that groups of transcripts accumulate together in morphological structures across evolution at rates higher than expected by chance. Together, our results demonstrate a relationship between transcript partitioning and organism morphology, independent from multicellularity, throughout diverse plant lineages.

## Introduction

**Figure 1:**
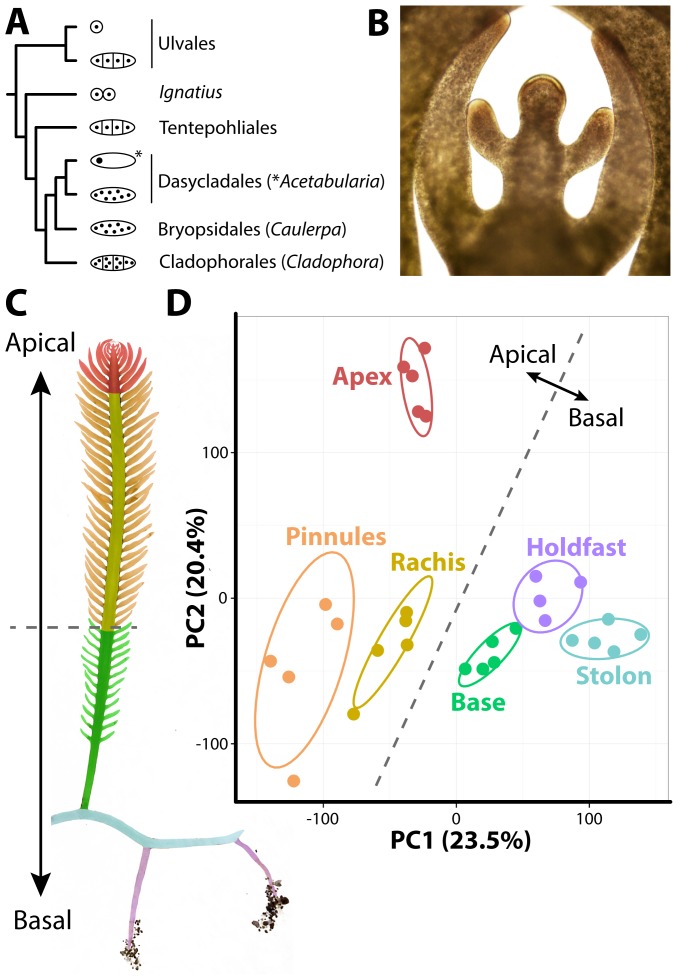
The evolutionary and developmental context of siphonous morphology in *Caulerpa taxifolia.* **A**) Evolutionary relationships among green algae with unicellular, siphonous, siphonocladous, and uninucleate multicellular lifestyles. Diagrams indicate body plan, redrawn from Cocquyt et al. [40]. **B**) The growing frond apex of *Caulerpa taxifolia*, producing young pinnules. **C**) Diagram of sampled regions. **D**) Principal Component Analysis (PCA) performed on organ RNA-Seq replicates based on transcript accumulation levels. 95% confidence ellipses are indicated for each sampled region. For convenience, a dotted line is provided separating apical from basal pseudo-organ types to relate data back to morphology. Colors indicate different sampled regions (as opposed to nodes in subsequent figures). Red, apex; orange, pinnules; yellow, rachis; green, frond base; blue, stolon; purple, holdfast.

Convergent morphologies have arisen multiple times in plants (Viridiplantae). Diverse cellular architectures underlie these moprhologies, with varying relationships between the number of nuclei per cell and the number of cells within an organism. Within the Chlorophyta, *Acetabularia* possesses an anchoring rhizoid, supporting stalk, and photosynthetic cap, but is, during most of its life cycle, a unicellular organism reaching heights of up to 10 cm with a single nucleus [1]–[3]. Another green alga, *Caulerpa*, is one of the largest known single-celled organisms, with stolons (up to meters in length) producing fronds and holdfasts [4]–[9] (**Fig. 1A–C**). Unlike *Acetabularia*, which is a single-celled organism, *Caulerpa* is coenocytic, with numerous nuclei. Siphonocladous chlorophytes have a chambered body plan compartmentalizing variable numbers of nuclei, as in *Cladophora*. Land plants (Embryophyta) are multicellular organisms, in which organs are composed of tissues and distinct cell types. Developmental biology in land plants was historically influenced by cell theory and studies in animals, in which organismal level morphology is an emergent property of cell division and histogenesis [10], [11]. Animal development is a poor example for land plants, in which morphogenesis is dissociated from histogenesis because cellular lineages and division patterns are largely independent from organ morphology.

For the above reasons, it has been argued [11] that cell theory is not as applicable to plants as in animals with respect to explaining how complex morphologies arise. In its place, Kaplan and Hagemann [11] argued for organismal theory, which they define as “[interpreting] the living protoplasmic mass as a whole, rather than considering its constituent cells as the basic unit.” In other words, the morphology in plants arises at the organismal level rather than as an emergent cellular property. Kaplan and Hagemann assert that “higher plants are also siphonous, but at a subtler, microscopic level.” Some of the siphonous features they argue land plants possess include: 1) cell division through a phragmoplast, 2) plasmodesmata, 3) the symplasm, 4) a multinucleate endosperm and megagametophytes, 5) distinct cytohistological zonations of the shoot apical meristem throughout the Embryophyta, 6) that cell lineage is often independent of morphology (e.g., in leaves), 7) and convergent morphology in multicellular red algae and land plants with different cell lineage patterns.

That land plants are truly siphonous is false: cell walls are a prominent features of land plants upon which morphology is dependent and land plants are multicellular organisms. However, it is useful to think about development in land plants from this unique perspective. That organ growth and morphogenesis are separate from cell division reduces the importance of cell-type specific transcript accumulation in these organisms. Transcriptomics and phylogenetics provide a mechanism to test hypotheses of cell versus organismal theory in siphonous green algae and land plants. Do the accumulation patterns of transcripts differ between single-celled and multicellular organisms with convergent morphology? Are groups of transcripts recurrently recruited to organs across large evolutionary distances regardless of whether an organism is multicellular?

Here, we provide a transcriptome of the giant coenocyte *Caulerpa taxifolia*. We detect a strong apical-basal gradient of transcript accumulation within the cell. Groups of transcripts with distinct functionalities accumulate in relevant pseudo-organs (morphological structures equivalent to a multicellular organ but not comprised of tissues or cells). Cell compartmentalization is partitioned in *Caulerpa*, despite its polynucleate condition, and transcripts are patterned according to the flow of genetic information, from transcription-to-translation in a basal-to-apical fashion. An analysis of the intersection of the transcriptomic atlases of a land plant (tomato, *Solanum lycopersicum*) and *Caulerpa* demonstrates a limited, recurrent recruitment of genes with similar functions to morphological structures. Our results provide a broad, evolutionary context for the relationship between the cell and organismal morphology at a molecular level within plants, confirming and expanding upon the organismal theory originally proposed by Kaplan and Hagemann [11].

## Results/Discussion

### Intracellular accumulation of transcripts

To develop a resource to address how organismal morphology can arise in the absence of multicellularity, we sequenced transcripts from multiple pseudo-organs and *de novo* assembled the intracellular transcriptome of *Caulerpa taxifolia* (see sequence submission information and **S1–S4 Datasets**). *Caulerpa taxifolia* stolons, upwards of 1 m in length, bear fronds (typically 15–30 cm long at maturity) with pinnately-arranged, tapered pinnules. The pinnules arise from active growth at the frond apex, which superficially resembles, in form and function, the apical cells and meristems of embryophytes (**Fig. 1B**). *Caulerpa taxifolia* is anchored by holdfasts, which take up phosphorus, nitrogen, and carbon from the substrate, and harbor both ecto- and endosymbiont bacteria that aid this process [9]. We sampled five replicates each of 1) the frond apex, 2) rachis, 3) pinnules, 4) the lower third of the frond base, 5) stolon, and 6) holdfast regions (**Fig. 1C**). One holdfast sample was lost when thawing for library preparation, reducing holdfast sampling to four replicates. The sample we sequenced was clonal in origin, having proceeded through numerous rounds of asexual reproduction. In its vegetative phase, *Caulerpa taxifolia* is a haplophasic diploid. *Caulerpa taxifolia* has one of the smallest genome sizes in its genus (∼100 Mbp, approximately the size of the *Arabidopsis thaliana* genome) and unlike other *Caulerpa* species does not exhibit extensive endopolyploidy [12], [13].

The transcriptome of *Caulerpa taxifolia* is dominated by patterning along the apical-basal axis. Throughout this manuscript, we use the terms “accumulation” and “abundance” in a relative sense to describe transcript accumulation patterns. Transcript accumulation in replicates derived from basal regions (holdfast, stolon, frond base) is highly similar and distinct from apical regions (frond apex, rachis, pinnules), as shown in a Principal Component Analysis (PCA) performed on replicates (**Fig. 1D**; **S5–S20 Datasets**). The growing frond apex in particular exhibits a unique transcriptomic signature, perhaps indicative of the “meristemplasm” found in this region, as previously described [5], [14].

**Figure 2:**
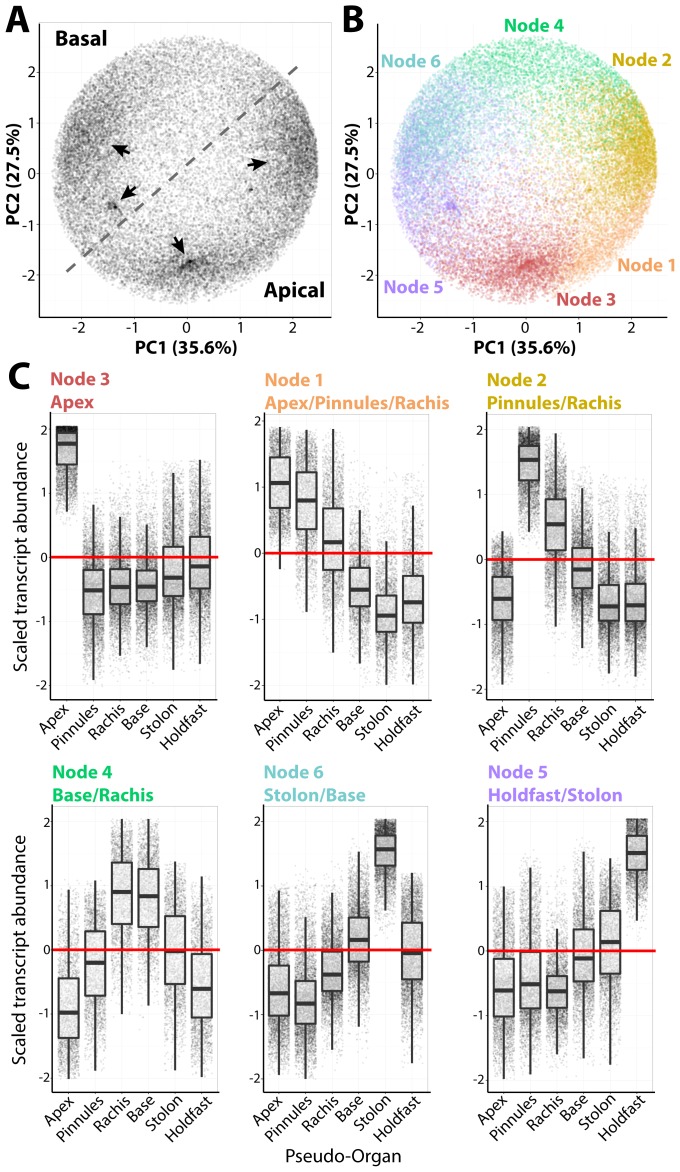
Intracellular accumulation of transcripts in a giant, single-celled organism. **A**) Principal Component Analysis (PCA) performed on transcript accumulation across sampled regions (the inverse of the PCA presented in **Fig. 1D**). Four major densities in the transcript accumulation variance structure are indicated by arrow. **B**) PCA was performed to visualize results of clustering by transcripts using Self-Organizing Maps (SOMs), visualized as different colors corresponding to nodes. **C**) Transcript accumulation profiles of genes belonging to different nodes, arranged with increasing abundance in an apical-to-basal direction. Scaled transcript abundance is such that the average abundance level across pseudo-organs for each transcript is 0 and variance is equal to 1. Scaled transcript abundance is shown as a boxplot and individual genes as jittered points (randomly displaced along the x-axis) to visualize transcript abundance distributions. Text for each node indicating those regions with scaled transcript abundance >0 is indicated.

A Self-Organizing Map (SOM) was used to partition transcripts into six clusters (nodes), each with a distinct accumulation pattern (**Figs. 2** **, S1**; **S21 Dataset**). These nodes explain prominent densities of transcripts with similar accumulation patterns across organs, as visualized using a PCA (**Fig. 2A–B**). The nodes are roughly organized along the apical-basal axis (**Fig. 2C**). For example, Node 3 transcripts exhibit high frond apex accumulation, and progressing basally to Node 5 transcripts which accumulate at high levels in the holdfast, nodes with intermediate accumulation patterns along the apical-basal axis are observed. The overall patterns of transcript accumulation, visualized using the combination of SOMs and PCA, can be explored for a random subset of genes in an interactive graphic we have prepared (http://danchitwood.github.io/CaulerpaGeneExpression/, for use with a Google Chrome web browser). Such strong apical-basal, intracellular partitioning of transcript accumulation is not surprising considering the influence of gravitropism on regeneration and anchoring [6], circadian movements of chloroplasts into and out of the apex, and cytoplasmic streaming along the frond and pinnule lengths [5].

Transcripts belonging to each node are highly enriched for associated Gene Ontology (GO) terms, often specific to cellular functions and organelles (**S22–S28 Datasets**). For example, Node 2 transcripts, which accumulate at high levels in the pinnules and rachis, are enriched for photosynthetic GOs, but additionally those associated with mitochondria, respiration, the electron transport chain and ATP synthesis, as well as the production of secondary metabolites. Node 3 transcripts with high abundances in the frond apex are enriched for COPI/II vesicle coat proteins and kinases. Most surprising is the overwhelming concentration of transcripts associated with nuclear functions—DNA replication and damage, chromatin, RNAi, and even the subunits of DNA polymerase II—in the frond base, stolon, and holdfast.

### Cell compartmentalization and morphology

Multicellular land plants possess an inherent constraint at the cellular level. Generally, every cell must have a nucleus, plastids, mitochondria, and cytoskeletal components to carry out basic metabolism, cell division, and differentiation, although numerous exceptions exist. But how is cellular compartmentalization distributed over similar morphology in *Caulerpa*? One hypothesis is that because morphogenesis is decoupled from multicellularity in *Caulerpa*, the distribution of different cell compartment identities might consolidate within distinct organs. That is, each cell is subcompartmentalized in a multicellular land plant, whereas the siphonous body plan of *Caulerpa* may maintain compartmentalization in pseudo-organs. Indeed, GO enrichment analysis reveals that transcript identity loosely follows the flow of genetic information progressing in a basal to apical direction in *Caulerpa* (**Fig. 2**, **S22–S28 Datasets**). Transcripts associated with transcriptional gene regulation accumulate at high levels in the holdfasts, stolon, and frond base, whereas those associated with translation are more abundant in the pinnules. Vesicular trafficking and kinase activity, associated with the cytoplasm and plasma membrane, are enriched within the frond apex.

**Figure 3:**
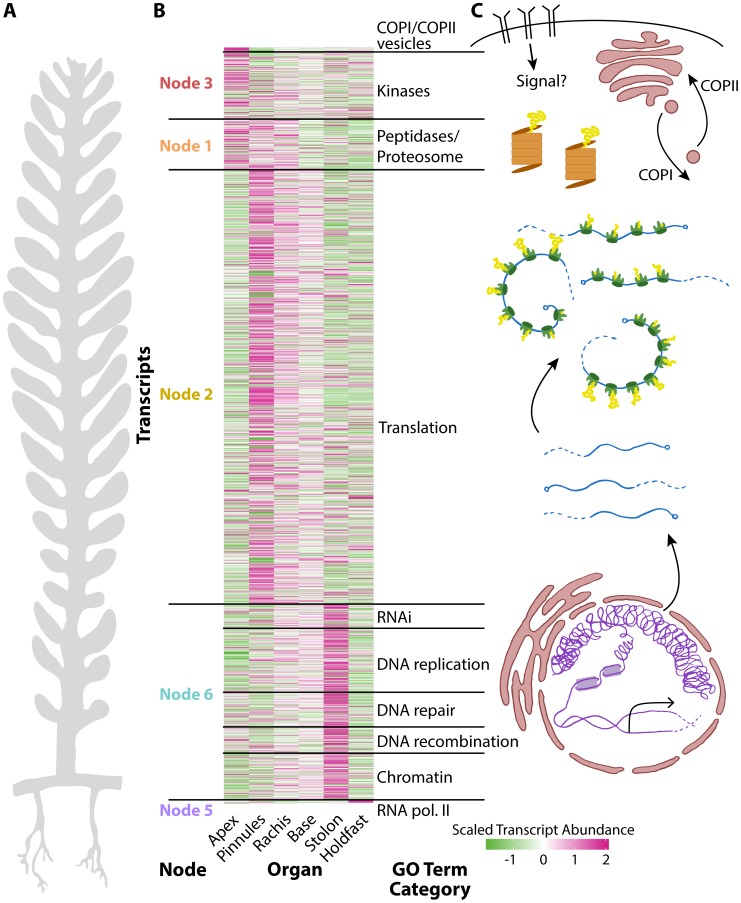
The relationship between cell compartmentalization and morphology. Panels within this figure correspond to each other, indicating a relationship between morphology, transcript accumulation, and cellular compartments. **A**) A diagram of *Caulerpa* morphology. Pseudo-organs roughly correspond to the apical-basal pattern of transcript accumulation shown in neighboring panel **B**) and the location of transcripts related to cellular compartments as shown in **C**). **B**) Heat map for genes belonging to select GO categories showing (left to right) node the parent GO term belongs to, transcript accumulation across pseudo-organs, and the general GO term category. Color indicates scaled transcript abundance, in which average transcript abundance is equal to 0 and variance equal to 1 for each transcript's abundance level across pseudo-organs. Green indicates low and magenta high scaled transcript abundance. **C**) Diagram of cellular compartments and the flow of genetic information from transcription to translation.

To explore the fundamental relationship between cell compartmentalization and organism morphology, we selected all transcripts belonging to significantly enriched GO terms related to transcriptional gene regulation, translation, and other important organellar and cell biological functions (**Fig. 3**, **S29 Dataset**). Transcripts encoding RNA polymerase II subunits are highly abundant in the holdfast. Those encoding numerous chromatin, epigenetic, DNA recombination, repair, and replication, and RNAi machinery components are highly abundant in the stolon. Transcripts related to translation accumulate at high levels in the photosynthetic tissues, mostly in the pinnules and somewhat in the rachis. Proteolysis transcripts are found in these regions too, but additionally in the frond apex where translational components accumulate at lower levels. COPI/II coat proteins and numerous kinases are highly abundant in the frond apex. The association of COPI/II trafficking with the apex, an active growth region containing white “meristemplasm,” is consistent with the previously reported enrichment of rough endoplasmic reticulum and Golgi bodies in this region [4], [5], [14].

The overall transcriptomic signature in *Caulerpa*—a single cell—is striking. From the holdfast to the frond apex, transcript accumulation loosely follows a nuclear-to-cytoplasm and transcriptional-to-translational pattern of identity (**Fig. 3**). The *Caulerpa* body plan is compartmentalized as if a single land plant cell, and different cellular compartments in *Caulerpa* are associated with different types of morphogenesis.

### Recurrent signatures of transcript accumulation underlying plant morphology

Land plant morphology, and the numerous and diverse morphologies of various chlorophytes, are derived from the monophyletic inheritance of a core gene set. In some instances, as between land plants and *Caulerpa*, convergent structures with related functions (for example, leaves and fronds, and roots and holdfasts) have evolved using these genes. If land plant morphology is viewed from the perspective of organismal theory proposed by Kaplan and Hagemann [11], and land plants are even considered to be siphonous and cellularization patterns arbitrary, then the accumulation of transcripts throughout the organism can be compared to detect molecular homology.

**Figure 4:**
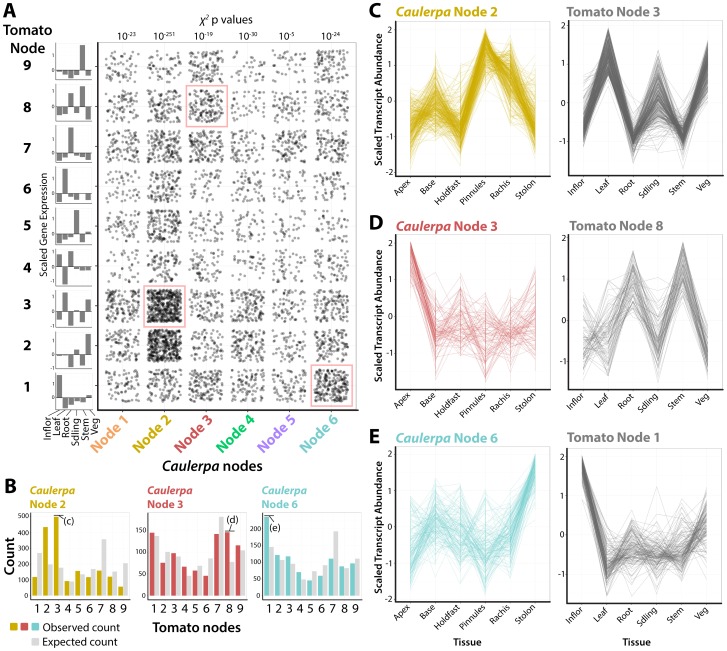
Recurrent recruitment of transcript accumulation to morphological structures in a land plant and *Caulerpa*. **A**) Intersection of transcript accumulation profiles by their *Caulerpa* node membership (x-axis) and corresponding accumulation pattern in tomato (*Solanum lycopersicum*). Each point corresponds to a *Caulerpa* transcript and its corresponding best BLAST-hit tomato homolog. χ**^2^** p values indicate probability of *Caulerpa* transcript membership among tomato nodes differing from expected null distribution. Along the side of the graph is indicated averaged scaled transcript abundance across sampled regions in tomato. Intersections of transcript accumulation detailed in **C–E** are indicated with red boxes. **B**) Bar graphs showing expected (gray) and observed (yellow, Node 2; red, Node 4; blue, Node 6) distributions of *Caulerpa* BLAST hits in tomato against tomato nodes. Intersections of transcript accumulation detailed in **C–E** are indicated. **C–E**) Line graphs of select intersections of transcripts with similar accumulation profiles in *Caulerpa*, the homologs of which are enriched for specific transcript accumulation patterns in tomato. Details of gene identities are discussed in the text. Inflor.  =  inflorescence, Leaf  =  leaf, Root  =  root, Sdling  =  seedling, Stem  =  stem, Veg  =  vegetative apex.

To what degree have similar transcript accumulation profiles been recruited to morphological structures in land plants and *Caulerpa*? To answer this question, we analyzed the intersection of the *Caulerpa* transcriptomic atlas (**Figs. 1** **–** **3**) with a transcriptomic atlas from a land plant (tomato, *Solanum lycopersicum* cv. M82) that was derived using similar molecular and bioinformatics methods as presented here [15]–[17]. Putative homologous transcripts from tomato (see Materials and Methods) were used to assign *Caulepra* transcripts to a corresponding tomato self-organizing map node [17] (**Fig. 4A**). The distribution of genes from each *Caulepra* node across tomato nodes was then compared to the expected distribution using a χ^2^ test. A higher than expected enrichment of genes assigned to a particular tomato node indicates that a group of *Caulerpa* genes with similar accumulation patterns are associated with a specific accumulation profile in tomato (**Fig. 4B**).

For example, *Caulerpa* Node 2 genes, which are highly abundant in the photosynthetically active pinnules and rachis (**Fig. 2**), are associated with tomato genes belonging to tomato Node 3, which are highly abundant in leaf, seedling, and vegetative apex samples (note: tomato and *Caulerpa* nodes are distinct and should not be confused with each other) (**Fig. 4B, C**). The intersection of *Caulerpa* Node 2 with tomato Node 3 is predominately photosynthetic genes (**S30 Dataset**). Although the molecular correspondence between photosynthetic structures is expected, other associations are less so. *Caulepra* Node 3 genes are highly abundant in the frond apex and are associated with tomato Node 8 genes that accumulate at high levels in the root and stem (**Fig. 4D**). Consistent with enriched GO terms in both *Caulerpa* and tomato, these genes are associated with vesicular trafficking (particularly COP cotamers) and vacuolar transporters (**S30 Dataset**). Interestingly, the *Caulerpa* Node 6 genes with high stolon abundance are associated with tomato Node 1 genes with high abundance in the inflorescence and relatively high abundance in the vegetative apex, both meristematic organs (**Fig. 4E**). The genes intersecting both nodes (**S30 Dataset**) are members of RNAi, chromatin, and DNA recombination, repair, and replication pathways, suggesting a molecular association between the stolon and meristems of land plants. The ability of the stolon to repetitively produce pseudo-organs (both fronds and holdfasts) and the enrichment of nuclear replication-associated transcripts indicates meristem-like identity at the molecular level.

The association between transcript accumulation profiles in *Caulerpa* and a land plant suggests, to a limited extent, molecular homology underlying morphology. Kaplan and Hagemann [11] argued that land plants, like *Caulerpa*, are siphonous. While the statement is extreme and not technically correct, reevaluating land plant development from this perspective is insightful, with respect to the role cells play in determining morphology. Morphogenesis and key patterning events in land plants rely on non-cell autonomous, symplastic movement of transcription factors and small RNAs, that transcend cell division patterns [18]–[21]. Spatially restricted transcripts in a siphonous organism, and their correspondence with land plant morphology, demonstrate that the plant form is achievable without cells and questions the centrality of cell division patterns in determining plant morphology.

## Materials and Methods

### RNA-seq library preparation, sequencing and preprocessing of reads

RNA-seq libraries were prepared from at least four replicates of the frond apex, rachis, pinnules, the frond base, stolon, and holdfast, sampling a prolifically growing *Caulerpa taxifolia* strain obtained from an aquarium in St. Louis, MO. The sampled strain was entrained to a circadian cycle using aquarium lighting roughly synchronized with the outside light-dark cycle. Sampling occurred mid-afternoon, at a time when chloroplasts were enriched in the frond apex (an important consideration, given the nightly retreat of chloroplasts into the frond base and stolon) [5], [22]. Large, intact fragments consisting of fronds, stolons, and holdfasts were removed from the marine aquarium and cleaned in synthetically prepared seawater for approximately 5 seconds to help reduce levels of outside contamination. Different samples corresponded to separately growing clones in the same aquarium. Samples were immediately immersed in liquid nitrogen after cleaning. The samples were then removed from the liquid nitrogen, dissected before they thawed, and placed into microcentrifuge tubes that were immersed again in liquid nitrogen. Samples were then stored at −80°C until library preparation. During thawing before library preparation, one holdfast microcentrifuge sample exploded and was removed from analysis, reducing the holdfast sample number to four. All five samples from other pseudo-organs were successfully processed.

Libraries were created using a custom high-throughput method for Illumina RNA-seq with a poly-A enrichment step [15], and sequenced in 100 bp paired-end format at the UC Berkeley Genomics Sequencing Laboratory on two lanes of HiSeq 2000 platform (Illumina Inc. San Diego, CA, USA). Library making was undertaken exactly as published in Kumar et al. [15] without modification of the protocol. We believe that the freezing step during sample preparation is important to bypass the *Caulerpa* wounding response for successful RNA isolation.

Reads were preprocessed using custom perl scripts that involved removal of low quality reads with average Phred quality score <20, trimming of low-quality bases with Phred score <20 from the 3′ ends of the reads, and removal of adapter/primer contamination. In addition identical reads, which originated during the PCR enrichment step of the library preparation, were collapsed into a single read using a custom perl script in order to reduce the computational resources required for transcriptome assembly. However, PCR-duplicated reads were retained for downstream quantification of transcript abundances. The pre-processed reads were sorted into individual samples based on barcodes using fastx_barcode_splitter and barcodes were trimmed using fastx_trimmer from Fastx_toolkit (http://hannonlab.cshl.edu/fastx_toolkit/). A total of 420 million reads (210 million paired-end 100×100), obtained after preprocessing, were used for transcriptome assembly.

### *De novo* transcriptome assembly and refinement

*De novo* transcriptome assembly was carried in a similar fashion as Ranjan et al. [23], but is described here again in detail. The Trinity software package (version r2013-02-16) was used to assemble, *de novo*, a *Caulerpa taxifolia* transcriptome using preprocessed RNA-seq reads [24]. The assembly was performed, using 24 large-memory computing nodes, at The Lonestar Linux Cluster at Texas Advance Computing Center (TACC, University of Texas). “Trinity.pl —seqType fq —JM 1000G —left reads-1.fq —right reads-2.fq —min_contig_length 200 —CPU 24 —bflyHeapSpaceMax 7G” was the command line used for assembly. Subsequently, assembly statistics and downstream analysis were performed in the iPlant atmosphere and Discovery computing atmosphere [25].

A total of 77,285 contigs with N50 (N50 is defined as the largest contig length such that using equal or longer contigs produces half the bases of the transcriptome) of 1243 bp, mean length of 807 bp and median of 433 bp, were assembled. In order to remove redundant and/or highly similar contigs, the contigs were then clustered using the CD-HIT-EST program from the CD-HIT suite at a sequence similarity threshold value (-c) of 0.95 and word-length (n) of 8, leaving other parameters at default [26]. This resulted in the final *Caulerpa* transcriptome assembly with 57,118 contigs and N50 of 813 bp, mean length of 632 bp and median of 381 bp (see sequence submission information). The prediction of likely coding sequences from 57,118 clustered contigs, using TransDecoder (http://transdecoder.sourceforge.net/), resulted in 35,827 putative open reading frames (ORFs)/coding sequences (CDS) (see sequence submission information).

### Functional annotation of transcriptome

The contigs from the final *Caulerpa* transcriptome assembly were compared to the NCBI nr (nonredundant) database (ftp://ftp.ncbi.nlm.nih.gov/blast/db/FASTA/nr.gz), *Arabidopsis* protein database (ftp://ftp.arabidopsis.org/home/tair/Sequences/blast_datasets/TAIR10_blastsets/TAIR10_pep_20110103_representative_gene_model_updated) and tomato (*Solanum lycopersicum*) ITAG2.3 protein database (ftp://ftp.solgenomics.net/tomato_genome/annotation/ITAG2.3_release/ITAG2.3_proteins.fasta) using BLASTX with an e-value threshold of 1e-5 (**S1 Dataset**) [27]. BLAST searches against the nr database resulted in annotation of 24,589 contigs (43% of clustered contigs) of which 20,146 contigs had reasonably stringent e-value of less than 1−e10. 17427 (13698 with e-value <1e−10) and 17392 (13274 with e-value <1e−10) contigs were annotated against *Arabidopsis* and tomato protein databases. When BLASTX comparison was performed against the nr database, more than 49% of annotated *C. taxifolia* contigs found top BLAST hits against the sequences from members of Cholorophyta, such as *Volvox carteri*, *Chlamydomonas reinhardtii*, *Chlorella variabilis*, and *Coccomyxa subellipsoidea*. Almost half of the top BLAST hits to cholorophytes confirm high sequence similarity between *C. taxifolia* and chlorophytes. The *Caulerpa* contigs were, further, compared explicitly against protein sequences of the chlorophytes *C. reinhardtii* and *V. carteri* using BLASTx, and vice-versa using tBLASTn with an e-value threshold of 1e−5 [27]. *Chlamydomonas reinhardtii* and *V. carteri* protein sequences were downloaded from Phytozome v10 (http://phytozome.jgi.doe.gov/pz/portal.html). BLAST searches against *V. carteri* and *C. reinhardtii* sequences found a hit for 19048 (33%) and 20377(36%) of assembled *Caulerpa* contigs, respectively (**S2 Dataset**). Reciprocal BLAST searches of *V. carteri* and *C. reinhardtii* sequences against *C. taxifolia* contigs found homologs for 42% of sequences of each species.

The BLASTX output generated against the NCBI nr database, with maximum twenty hits for each contig, was used for Blast2GO analysis to annotate the contigs with GO terms describing biological processes, molecular functions, and cellular components [28]. Blast2GO performs GO annotation by applying an annotation rule (AR) on the found ontology terms from the BLAST-hits, which is based on annotation score. The default e-Value hit filter (1e−6) and annotation cut-off (55) was used to calculate the annotation score. Our gene ontology annotations are, by necessity, based in part on the inclusion of hits using a relatively low e-value threshold. It will be critical in the future to validate these assignments using functional analysis. After the Blast2GO mapping process, proper GO terms were generated followed by use of ANNEX and GO Slim, which are integrated in the Blast2GO software, to enrich the annotation (**S3, S4 Datasets**). Sequence descriptions were also generated from Blast2GO, which are arbitrary nomenclature based upon degrees of similarities identified in the nr database according to e-value and identity to blasted genes (**S1, S2 Datasets**). BLASTX against the nr database resulted in annotation of 24,589 contigs (43% of clustered contigs) of which only 14,206 (25% of clustered contigs) were assigned GO-terms. Given the problems associated with the *de novo* transcriptome assembly algorithms and lack of functional tools in *Caulerpa*, BLASTX annotation of 43% of clustered contigs and GO annotation of only 25% of clustered contigs is not surprising. Similar functional annotation for only a fraction of assembled contigs has been noted for other *de novo* assembled plant transcriptomes [23], [29], [30]. These non-annotated contigs likely correspond to 3′ or 5′ untranslated regions, non-coding RNAs, or short sequences not containing a known protein domain, some of which might represent potential *Caulerpa*-specific genes.

### Mapping reads to contigs and normalized count data

RSEM (RNAseq by expectation maximization), which allows for an assessment of transcript abundances based on the mapping of RNA-seq reads to the assembled transcriptome, was used for transcript abundance estimation of the *de novo* assembled transcripts [31]. Due to read mapping ambiguity among *de novo* assembled transcripts, it is common to have the same read mapped to multiple contigs. RSEM models the reads mapped at multiple contigs taking into account length of target contigs, number of mismatches, sequencing errors, etc., and generates an estimated read count for each contig. Single end reads, retaining the PCR-duplicated reads, from individual libraries of each *Caulerpa* sample were mapped to clustered contigs using the perl script run_RSEM_align_n_estimate.pl that employs RSEM, followed by joining RSEM-estimated abundance values for each sample using merge_RSEM_frag_counts_single_table.pl, generating raw estimated counts for each contig from each *Caulerpa* sample (**S5 Dataset**). Subsequently, differential expression analysis for each organ pair was carried out using run_DE_analysis.pl, which involves the Bioconductor package EdgeR in the R statistical environment [32]. Contigs that had RSEM-estimated counts ≥30 for all samples combined were used for transcript abundance estimates. Normalization factors were calculated using the trimmed mean of M-values method to obtain normalized read count per million for each contig of a sample. This normalized reads per million was then used for the pair-wise differential expression analysis for each organ pair using EdgeR. The lists of significant differentially expressed contigs (FDR<0.05) for each organ-pair comparison are presented in **S6–S20 Datasets**. All the Perl scripts used for read mapping, generating read counts and differential expression analysis are documented with Trinity software suite [33].

### Principal Component Analysis (PCA), Self Organizing Maps (SOM) clustering, and other statistical analyses

Those transcripts differentially expressed between at least one organ pair (**S6–S20 Datasets**) were subsequently used to find clusters of genes with similar transcript accumulation patterns using Self Organizing Maps (SOMs) [34]. Differentially expressed transcripts were averaged across replicates for each pseudo-organ sample. Averaged transcript abundance values were then scaled across pseudo-organs to arrive at scaled transcript accumulation patterns which were used to assign cluster membership. Scaling was performed using the scale() function in the base package using default settings, such that the average transcript abundance value across pseudo-organs was 0 and the variance equal to 1. To cluster transcripts across organs, a 3×2 hexagonal SOM was implemented, using the Kohonen package in R [35], [36]. 100 training iterations were used during clustering, over which the α-learning rate decreased from 0.05 to 0.01. Mean distance of transcript accumulation patterns to their closest unit stabilized after approximately 15 iterations of training.

A decision to use six clusters was arrived at by first analyzing the results of a Principal Component Analysis (PCA) on scaled transcript accumulation across tissues, using the prcomp() function in R with default settings. Average and scaled transcript accumulation levels across organs, as well as SOM cluster membership and PC values are provided (**S21 Dataset**). Four main densities in the variance attributable to accumulation patterns were discernable (arrows, **Fig. 2A**), and the results of a 4 cluster SOM largely overlap with the densities. Variance in accumulation among transcripts across organs was large, however, and the decision to specify 6 SOM clusters not only produced clusters with unique accumulation patterns and lower variance in abundance (**Fig. 2C**), but also yielded clusters with more interpretable GO enrichment categories (that is, significant GO enrichment consistent with known biology, such as photosynthetic GOs enriched in nodes with high pinnule transcript abundance). To verify that 6 GOs was indeed the maximum cluster number specifying unique transcript accumulation profiles without redundancy, we undertook partitioning of the PCA space into a variable number of SOM clusters over 100 iterations for each node number with random seeds. Linear Discriminant Analysis (LDA) was performed on genes maximizing separation of cluster identity using PCs 1–5 (PC6 explained negligible amounts of variance and could not be incorporated into the LDA) using the lda function from the MASS package [37]. The predict function (stats package) and table function (base package) were used to reallocate genes to predicted clusters (within MASS) using the linear discriminants. A high fraction of a node's originally assigned transcripts by SOM being reassigned correctly indicates little redundancy in node transcript accumulation patterns. Starting with 6 nodes, reassignment using LDA begins to drop before reaching a plateau of low reassignment rates, indicating that choosing 6 nodes maximizes the number of unique accumulation profiles represented by clusters without redundancy (see **S1 Fig.** for results).

Analysis of intersection between tomato and *Caulerpa* transcriptomic atlases was undertaken using data from Chitwood et al. [17]. Best BLASTX hits of *Caulerpa* transcripts to tomato (*Solanum lycopersicum*) (see “Functional annotation of transcriptome” above and **S1, S2 Datasets**) were used to assign tomato transcript accumulation patterns, across a number of organs, to *Caulerpa* transcript accumulation patterns. The distribution of tomato transcripts assigned to tomato SOM nodes was taken as the null distribution and compared to the number of *Caulerpa* transcripts assigned to each tomato node. p values, indicating the degree of significant difference between the two distributions, were obtained from χ^2^ values using the chisq.test function (stats package).

Clusters of transcripts were analyzed for GO enrichment terms at a 0.05 FDR cut-off using the “goseq” package in Bioconductor (**S22–S28 Datasets**) [38]. Unless otherwise specified, all statistical analyses on transcript accumulation were performed using R [36] and data visualization using the ggplot2 package [39].

### Sequence submission

The quality filtered, barcode-sorted and trimmed short read dataset, which was used for transcriptome assembly, was deposited to the NCBI Short Read Archive under accessions SRR1228213–SRR1228223, SRR1228225–SRR1228232, SRR1228234–SRR1228238 and. SRR1228240–SRR1228244. All assembled contigs have been deposited at DDBJ/EMBL/GenBank under the accession GBCY00000000. The version described in this paper is the first version, GBCY01000000.

Sequences of all contigs of Caulerpa_final_transcriptome, obtained after clustering of transcripts, can be downloaded as a FASTA file at http://de.iplantcollaborative.org/dl/d/80CF0D47-5A80-4CE7-B6DF-F4A7ED803493/Caulerpa_final_transcriptome.fasta. The contigs were named as Ctaxi_contig plus a serial number with the Trinity identifiers.

Sequences of all predicted ORFs from the *Caulerpa* transcriptome assembly can be downloaded as a FASTA file at http://de.iplantcollaborative.org/dl/d/40273882-35DD-4930-9DBD-6D60CEAA7890/Caulerpa_predicted_ORFs.fasta. The ORFs were named as Ctaxi_predicted_CDS plus a serial number.

## Supporting Information

S1 Fig Selection of Self-Organizing Map (SOM) cluster number. A graph showing results of partitioning of Principal Component Analysis (PCA) space into the indicated number of clusters using SOMs followed by the use of Linear Discriminant Analysis (LDA) to determine the discernibility of the resulting SOM clusters. For each selection of cluster number, SOM clustering was performed 100 times using random seeds, followed by determining the fraction of transcripts from each determined cluster that could be successfully reassigned using linear discriminants. See Materials and Methods for details. Y-axis: distributions of successful reassignment rates for clusters determined over 100 SOM partitionings. X-axis: the number of SOM clusters PCA space is partitioned into and the topology of the SOM.
(TIF)

S1 Dataset BLASTX annotation of contigs and comparison against *Arabidopsis* and tomato protein databases. Combined annotation of contigs from the *Caulerpa* transcriptome assembly obtained from BLASTX against non-redundant (nr), *Arabidopsis* TAIR10, and tomato ITAG2.3 protein databases. The annotation includes sequence descriptions from Blast2GO, top hit descriptions to the nr database, e-values for the top hit to the nr database, and gene ID, gene name and description, and e-values for the top hits to *Arabidopsis* and tomato databases.
(TXT)

S2 Dataset BLASTX annotation of contigs and comparison against other green algae protein databases. Annotation of *Caulerpa* contigs obtained from BLASTX against *Chlamydomonas reinhardtii* and *Volvox carteri* protein databases. The annotation includes gene ID, gene description and e-value for the top hits to *C. reinhardtii* and *V. carteri* databases.
(TXT)

S3 Dataset Complete GO annotation of contigs. Gene Ontology (GO) description for all annotated *Caulerpa* contigs using complete GO-identifiers.
(TXT)

S4 Dataset GO slim annotation of contigs. Gene Ontology (GO) description for all annotated *Caulerpa* contigs using GO slim identifiers.
(TXT)

S5 Dataset RSEM-estimated counts for contigs. RSEM-estimated raw read counts, that are adjusted for the reads mapping to more than one contig, for each contig from all replicates of each *Caulerpa* pseudo-organ. These read counts were used for TMM normalization and subsequent differential expression analysis.
(TXT)

S6 Dataset Differentially expressed transcripts between the frond apex and frond base. Differentially expressed transcripts (FDR<0.05) and their annotation information are provided.
(TXT)

S7 Dataset Differentially expressed transcripts between the frond apex and holdfast. Differentially expressed transcripts (FDR<0.05) and their annotation information are provided.
(TXT)

S8 Dataset Differentially expressed transcripts between the frond apex and pinnules. Differentially expressed transcripts (FDR<0.05) and their annotation information are provided.
(TXT)

S9 Dataset Differentially expressed transcripts between the frond apex and rachis. Differentially expressed transcripts (FDR<0.05) and their annotation information are provided.
(TXT)

S10 Dataset Differentially expressed transcripts between the frond apex and stolon. Differentially expressed transcripts (FDR<0.05) and their annotation information are provided.
(TXT)

S11 Dataset Differentially expressed transcripts between the frond base and holdfast. Differentially expressed transcripts (FDR<0.05) and their annotation information are provided.
(TXT)

S12 Dataset Differentially expressed transcripts between the frond base and pinnules. Differentially expressed transcripts (FDR<0.05) and their annotation information are provided.
(TXT)

S13 Dataset Differentially expressed transcripts between the frond base and rachis. Differentially expressed transcripts (FDR<0.05) and their annotation information are provided.
(TXT)

S14 Dataset Differentially expressed transcripts between the frond base and stolon. Differentially expressed transcripts (FDR<0.05) and their annotation information are provided.
(TXT)

S15 Dataset Differentially expressed transcripts between the holdfast and pinnules. Differentially expressed transcripts (FDR<0.05) and their annotation information are provided.
(TXT)

S16 Dataset Differentially expressed transcripts between the holdfast and rachis. Differentially expressed transcripts (FDR<0.05) and their annotation information are provided.
(TXT)

S17 Dataset Differentially expressed transcripts between the holdfast and stolon. Differentially expressed transcripts (FDR<0.05) and their annotation information are provided.
(TXT)

S18 Dataset Differentially expressed transcripts between the pinnules and rachis. Differentially expressed transcripts (FDR<0.05) and their annotation information are provided.
(TXT)

S19 Dataset Differentially expressed transcripts between the pinnules and stolon. Differentially expressed transcripts (FDR<0.05) and their annotation information are provided.
(TXT)

S20 Dataset Differentially expressed transcripts between the rachis and stolon. Differentially expressed transcripts (FDR<0.05) and their annotation information are provided.
(TXT)

S21 Dataset Averaged and scaled transcript abundance levels, principal component values, and assigned self-organizing map nodes. Provided are mean transcript abundance values of transcripts differentially expressed across pseudo-organs (indicated by “mean” followed by the pseudo-organ name), scaled transcript abundance values across pseudo-organs (indicated by “sc” followed by the pseudo-organ name), principal component values (PCs 1–6) resulting from a Principal Component Analysis (PCA) on transcript accumulation across pseudo-organs, assigned Self-Organizing Map cluster (“node”), and distance of transcript accumulation profile from assigned cluster (“distance”).
(TXT)

S22 Dataset GO terms enriched for transcripts belonging to Node 1. IDs and descriptions for significantly enriched GO terms.
(TXT)

S23 Dataset GO terms enriched for transcripts belonging to Node 2. IDs and descriptions for significantly enriched GO terms.
(TXT)

S24 Dataset GO terms enriched for transcripts belonging to Node 3. IDs and descriptions for significantly enriched GO terms.
(TXT)

S25 Dataset GO terms enriched for transcripts belonging to Node 4. IDs and descriptions for significantly enriched GO terms.
(TXT)

S26 Dataset GO terms enriched for transcripts belonging to Node 5. IDs and descriptions for significantly enriched GO terms.
(TXT)

S27 Dataset GO terms enriched for transcripts belonging to Node 6. IDs and descriptions for significantly enriched GO terms.
(TXT)

S28 Dataset Annotation of genes belonging to significantly enriched GO terms for each node. Provided are annotation details for those genes belonging to GO terms significantly enriched for each node.
(TXT)

S29 Dataset GO term categories. Provided are categories used to classify similar GO terms that are used in Fig. 3.
(TXT)

S30 Dataset Intersection of *Caulerpa* and tomato (*Solanum lycopersicum*) transcriptomic atlases. Provided are *Caulerpa* contig IDs (“seq_id”), their best BLASTX tomato hit from the tomato ITAG2.3 transcriptome version (“itag”), the node the *Caulerpa* contig belongs to (“caulerpa_node”), the node the tomato best hit belongs to (“tomato_node”), scaled transcript abundance across *Caulerpa* pseudo-organ samples (“ct” preceding pseudo-organ name), scaled transcript abundance across tomato organs (“sl” preceding organ name), percent identity (“X_percidentity”), e values (“X_eVal”), bit scores (“X_bitScore”), the ITAG2.3 description (“ITAG2.3_hit_description”), and information concerning the best *Arabidopsis* TAIR hit to the respective ITAG2.3 tomato transcript.
(TXT)
