# Supplementary figures and images for: An Intracellular Transcriptomic Atlas of the Giant Coenocyte Caulerpa taxifolia
Source: PLoS Genet. 2015 Jan 8;11(1):e1004900. doi: 10.1371/journal.pgen.1004900 (PMC4287348; doi:10.1371/journal.pgen.1004900)

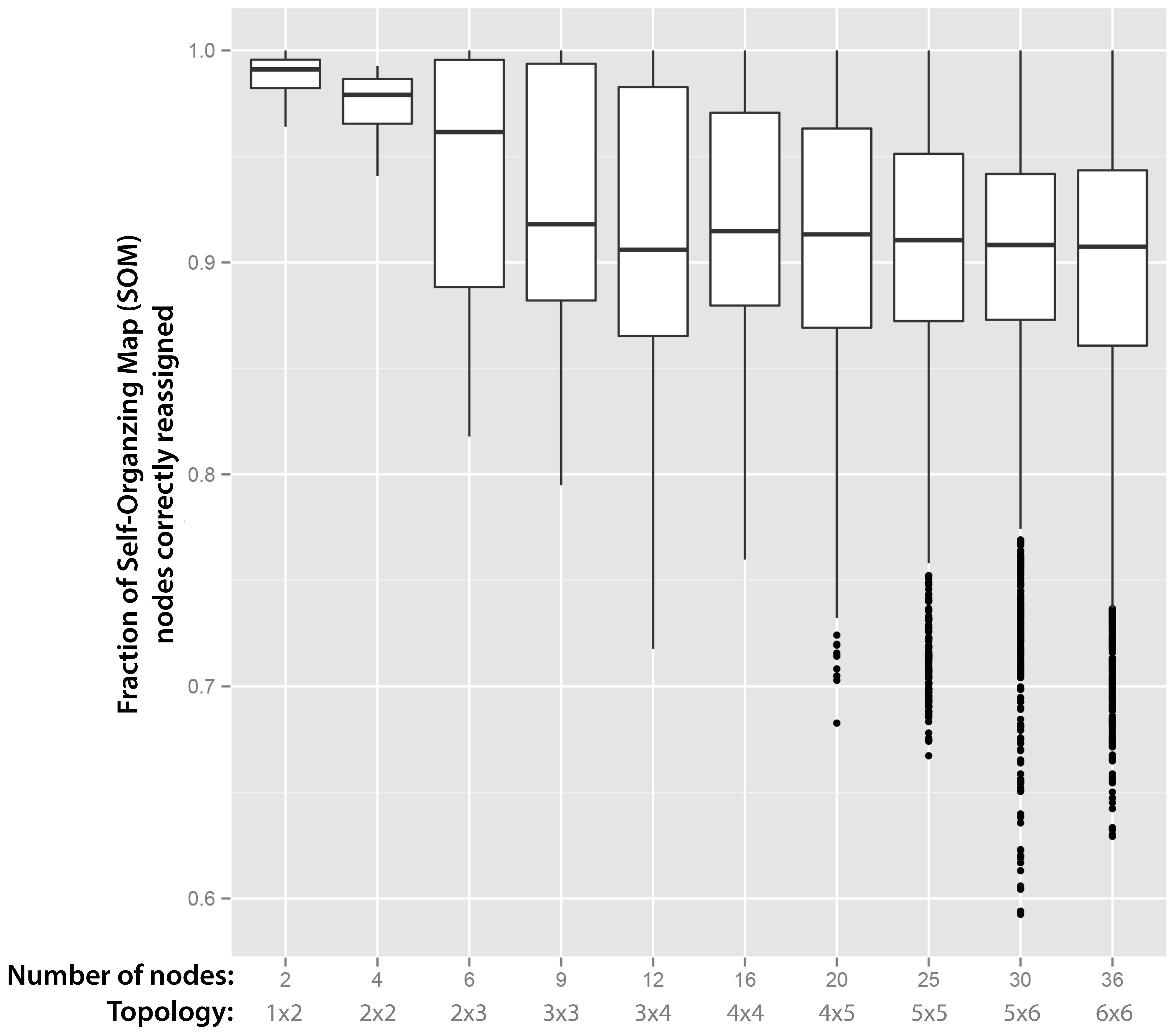

Supplement: S1 Fig — Selection of Self-Organizing Map (SOM) cluster number. A graph showing results of partitioning of Principal Component Analysis (PCA) space into the indicated number of clusters using SOMs followed by the use of Linear Discriminant Analysis (LDA) to determine the discernibility of the resulting SOM clusters. For each selection of cluster number, SOM clustering was performed 100 times using random seeds, followed by determining the fraction of transcripts from each determined cluster that could be successfully reassigned using linear discriminants. See Materials and Methods for details. Y-axis: distributions of successful reassignment rates for clusters determined over 100 SOM partitionings. X-axis: the number of SOM clusters PCA space is partitioned into and the topology of the SOM. (TIF) [file pgen.1004900.s001.tif]
